# COVID-19, Livestock Systems and Food Security in Developing Countries: A Systematic Review of an Emerging Literature

**DOI:** 10.3390/pathogens10050586

**Published:** 2021-05-11

**Authors:** Assem Abu Hatab, Lena Krautscheid, Sofia Boqvist

**Affiliations:** 1 Department of Economics, Swedish University of Agricultural Sciences, 750 07 Uppsala, Sweden; Lena.Krautscheid@t-online.de; 2 Department of Economics & Rural Development, Arish University, 455 11 Arish, Egypt; 3 Department of Biomedical Science and Veterinary Public Health, Swedish University of Agricultural Sciences, 750 07 Uppsala, Sweden; sofia.boqvist@slu.se

**Keywords:** COVID-19, livestock systems, food security, developing countries, systematic literature review

## Abstract

In this paper, we carried out a systematic literature review to document the emerging scientific knowledge about COVID-19 impact on livestock systems and food security in developing countries to identify gaps and possible avenues for future research undertakings. Specifically, we systematically reviewed 68 peer-reviewed articles extracted based on rigorous selection criteria from Scopus, PubMed and ISI Web of Science databases and published between December 2019 and February 2021. Our results reveal that livestock supply chains presented an important ‘intermediary’ pathway through which the pandemic affected various dimensions of food security in developing countries. Although the research response has been rapid in terms of both quantity and temporal succession, we find a highly suggestive disjunction in studies analyzing the interconnections between COVID-19 pandemic, livestock systems and food security in developing countries. With respect to the livestock supply chain, the bulk of the reviewed evidence focuses on production and consumption, whereas considerably less focus is given to the pandemic’s impact on intermediaries within livestock chains, including traders, intermediaries and processors. The analysis of livestock supply chain resilience revolves predominantly around the ‘absorbance’ and ‘recovery’ phases of resilience, whereas only a small subset of the literature investigates actions taken by supply chain actors to ‘plan’ or to ‘adapt’ livestock systems in order to reduce their vulnerability and enhance their overall resilience. Furthermore, food security has often been narrowly defined, with the majority of articles focusing on ‘availability’ and ‘accessibility’ to food due to the pandemic, and other dimensions of food security, including utilization, stability and sustainability, have been widely neglected. Based on our findings, we recommend future research to examine the dynamics of propagation of COVID-19 impact through livestock supply chains in order to develop more targeted interventions that enhance the capacity of developing countries to cope with this and future disruptions and mitigate their food insecurity outcomes. To this end, more holistic, integrated and resilience-based approaches are much recommended to recognize the complex nature of livestock systems in developing countries and to address the multifaceted and widespread effects of COVID-19 on food security channeled through livestock chains.

## 1. Introduction

The outbreak of the COVID-19 pandemic starting in 2020, together with the subsequent measures adopted by governments in developing countries to contain the spread of the virus, have exerted substantial impact on livestock production systems [1]. The FAO [2] reveals that the COVID-19 pandemic has tremendous effects on livestock systems, with production especially evoked by the restrictions on human mobility, leading to shortages in farm labor and subsequently causing unequalled challenges to transportation, processing, retailing and other logistics, and momentous shifts in consumer demand [3]. In particular, small livestock producers in developing countries, who represent the majority of producers, have been affected the most by the pandemic because of the small-scale and the informal nature of their activities [4]. Therefore, there is growing evidence that the impact of COVID-19 on livestock systems in developing countries threatens to further undermine livelihoods and worsen the food insecurity of poor and rural households in developing countries [1]. 

The strong interlinkages between livestock systems and food security in the context of developing countries are attributive to the significant contributions of livestock supply chains directly to the supply of livestock products and the consumption and nutrition outcomes of livestock-source foods, and indirectly to employment, livelihoods and overall sustainable development [5]. In this respect, previous studies link livestock production and consumption of livestock-source food to improved food security status and enhanced physical and mental health outcomes, particularly among women and children [6,7]. In addition, livestock systems represent at least one-third of agricultural gross domestic production in developing countries and contribute significantly to employment and livelihoods of the poor and smallholder livestock keepers [8]. Furthermore, livestock acquisition is widely regarded as a means of income generation and diversification, a financial instrument and a pathway out of poverty [9]. Therefore, it is argued that solutions for many of the food security challenges facing developing countries lie in how livestock are raised and managed [10]. 

Under the dynamics of the COVID-19 pandemic, livestock systems in developing countries face additional pressures that magnify the impact of other already-existing challenges (e.g., climate change, urbanization and demographic changes and changing dietary preferences) that constrain their capacity to foster food and nutrition security [1,4,11]. A quick look at the literature on previous epidemics and livestock systems reveals that outbreaks of diseases and epidemics have always been associated with disruptions in livestock supply chains and major socioeconomic consequences on actors within these chains [2]. For example, over the last two decades, disease outbreaks such as the Middle East Respiratory Syndrome (MERS), Severe Acute Respiratory Syndrome (SARS), High Pathogenic Avian Influenza, Ebola and Zika have had major consequences on food security in developing countries [12]. In particular, these disease outbreaks have severely affected livestock supply chains in multiple ways, compounding food insecurity outcomes by decreasing livestock supply and productivity [12], deteriorating the safety and quality of livestock commodities [13,14], decreasing employment in livestock sectors and livelihoods [15,16], reducing consumer demand and purchasing power [17,18] and increasing food loss and waste [9]. 

In the wake of the emergence of the COVID-19 pandemic in early 2020, a growing body of research has been published on its impact on crop and livestock systems, and on food security in developed and developing countries. In this context, Bobrowski et al. [19] point out that COVID-19 has caused the quickest research response to a pandemic in recent decades and stimulated research on its various effects, including on livestock systems and food security. Considering the importance of livestock systems to food security and nutrition in developing countries, as well as the projections that pandemics like COVID-19 will happen more frequently in the future [20], this study systematically reviews the peer-reviewed literature on COVID-19, livestock systems and food security in developing countries to characterize and synthesize the current understanding on their interlinkages and identify priorities for future research. Specifically, we reviewed the state of evidence on this topic during the period from December 2019 to February 2021, with the aim to answer two key questions: (a) how has the COVID-19 pandemic affected livestock systems in developing countries, and (b) what are the consequences of these effects on various dimensions of food security in developing countries?

Answers to these questions are anticipated to make three main contributions to the literature. First, the uniqueness of the COVID-19 pandemic and its unprecedented effects on livestock systems make it incomparable with previous disease outbreaks [21,22] and necessitate tailored diagnosis and adaptation strategies to mitigate its consequences on food security. A systematic literature review (SLR) at this early stage of literature development, when many research undertakings are being designed or initiated, can identify key research gaps, unresolved questions and avenues for future research in this area. Second, while the effects of COVID-19 on food supply chains in general have prompted growing researchers’ attention in the past few months, there is very little evidence on the full direct and indirect impact of the pandemic on livestock supply chains [23] as well as a lack of research based on the experiences of livestock producers and consumers in developing countries [24]. In this respect, Grace et al. [25] point out that despite the fact that livestock systems in developing countries have experienced several extreme events in recent years, the ability of the livestock sectors to predict, prevent and control these events is limited and thus there is a need to direct research activities to specific topics of relevance to the context of livestock systems in these countries to implement interventions that enhance their preparedness to future pandemics.

A third contribution of this paper lies in the adoption of a system approach to characterize the literature in relation to the three components of focus: COVID-19, livestock systems and food security, by recognizing that livestock systems constitute multiple activities and actors, including both those who are directly engaged in the livestock chain and those who form the environment in which the livestock chain exists. This approach offers the opportunity to lay out the linkages and relationships of these components, which can contribute to developing and implementing mitigation strategies and management interventions to build resilience of livestock systems, enhance their preparedness to future pandemics and improve their capacity to foster food security in developing countries.

To answer the two questions of this SLR and to identify the interlinkages between the COVID-19 pandemic, livestock systems and food security in the context of developing countries, we define a livestock system as the chain of livestock activities ‘taking place in a specific geographical context in which livestock-source food is consumed, connecting livestock production, processing, distribution, consumption and waste management, as well as the associated regulatory institutions and activities’ [26]. With regard to food security, we adopt the definition of the FAO [27] stating that food security encompasses four traditional dimensions, namely, food security availability, access, utilization and stability, and is a situation that exists when people have ‘secure access to sufficient amounts of safe and nutritious food for normal growth and development and an active and healthy life’, and that it traditionally has four main dimensions: availability, accessibility, utilization and stability.

## 2. Systematic Literature Review Approach

### 2.1. Search Codes

This SLR began with an ad-hoc literature review using Google Scholar and Google to develop a broad conceptualization of what is known and which emergent issues require further investigation in relation to the impact of COVID-19 on livestock systems and food security in developing countries. This process yielded a number of relevant peer-reviewed articles that were used to formulate the search string and identify the most relevant search codes. The original list of search codes were sent to members of Swedish University of Agricultural Science (SLU) Global Network, a university based network involving over 250 researchers whose research interests focus on sustainability of agrifood systems in developing countries and who are actively engaged in research collaborations with partners in developing countries. Specifically, members of this network received four original lists of keywords related to ‘COVID-19 pandemic’, ‘livestock systems’, ‘food security’ and ‘developing countries and regions’. Then, they were asked to rate the relevance of each keyword on a Likert-scale ranging from 1 (= not relevant at all) to 5 (= extremely relevant). In addition, they were allowed to suggest deletions, revisions or inclusions of additional search codes. The feedback provided by this group of experienced researchers was used to validate, refine and develop a final list of the most relevant search terms for the objective of this SLR. As shown in Table S1 in the attached Supplementary Materials, the final list of search terms consisted of 11 search codes relating to ‘COVID-19 pandemic’, 44 search codes relating to ‘livestock systems’, 36 search codes relating to ‘food security’ and 92 search codes relating to ‘developing countries and regions’. 

### 2.2. Search Strategy 

Prior to the implementation of the bibliographic databases’ searches, members of the research team who participated in the search process were trained by experienced librarians and researchers in conducting systematic literature reviews and databases search. The search strategy and eligibility criteria in this SLR were guided by the Preferred Reporting Items for Systematic Reviews and Meta-Analyses (PRISMA) [28], which consist of four consequent phases that describe the identification, eligibility, screening and inclusion criteria of the article that fall under the scope of this SLR. The review covered peer-reviewed publications on COVID-19 pandemic, livestock systems and food security in developing countries published online between December 2019 and February 2021 (The authors relied on the UN’s World Economic Situation and prospects 2021 to identify a list of developing countries (https://www.un.org/development/desa/dpad/publication/world-economic-situation-and-prospects-2021/, accessed on 26 February 2021). These publications were extracted from three major online academic literature databases: PubMed, ISI Web of Science and Scopus. As noted by Falagas et al. [29], these bibliographic databases have wider coverage, a good reputation and advanced exportability features, and allow for flexible formulations of search strings.

**Figure 1:**
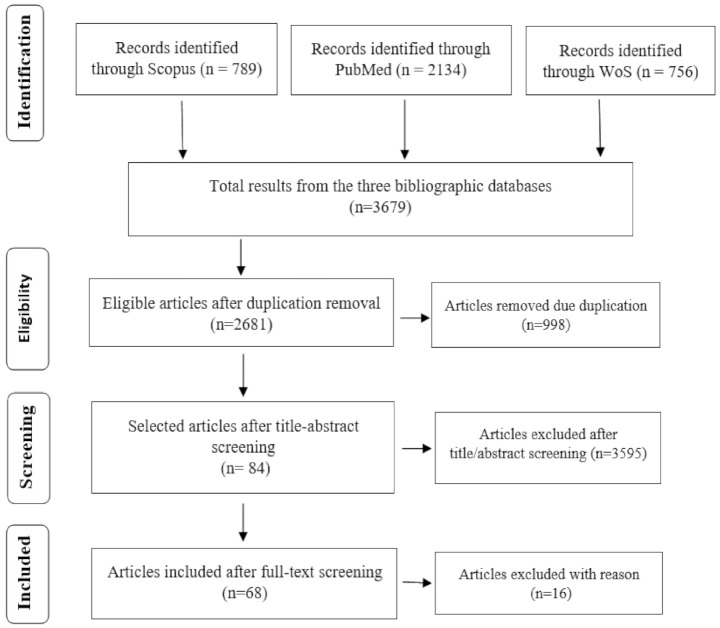
Selection process of the reviewed articles based on the inclusion criteria.

In addition to the ad-hoc literature review that was conducted to identify search codes, our search strategy consisted of two main steps. In the first ‘pilot’ step, we implemented the review protocol in Figure 1 to further refine the review protocol and test our inclusion criteria, test quality assessment procedures and design a data extraction form. The process and results of this pilot search were discussed with two experienced librarians. Next, the ‘actual’ systematic search of the three bibliographic databases was performed. As shown in Figure 1, this step yielded a set of 3679 candidate peer-reviewed articles, distributed as follows: Scopus (789 articles = 21.4% of the total candidate articles), PubMed (2134 articles = 58% of the candidate articles) and WoS (756 articles = 20.6% of the total candidate articles).

### 2.3. Inclusion Criteria and Quality Control Procedures

Expectedly, the bibliographic databases’ search yielded a large number of candidate articles, including many duplicates and articles that were irrelevant to the scope of this SLR. To filter these results, a duplicate removal exercise was performed, leading to the exclusion of 998 articles. Next, a title and abstract screening was conducted, where articles were included only if (i) the title was related to the first three categories of our search codes (that is, COVID-19, food Security, Livestock Systems) and (ii) the focus of the candidate articles was on any of the countries regions listed under category 4 (developing countries) in Table S1 in the Supplementary Material. Since the title and abstract screening was performed by three different members of the research team, a quality control exercise was implemented in order to ensure a common understanding by members of the research team of the inclusion and exclusion criteria. Specifically, the three researchers were asked to re-screen a sub-sample of 5% of the candidate articles resulting from each bibliographic database. That is, each article within this sub-sample was examined by the three members of the research team. The results of this exercise indicated an overall similarity index of 94.7%. Based on the results of duplicate removal and title and abstract screening, 84 articles were advanced to the full-text screening phase. 

During the full-text screening phase, a final decision regarding the inclusion of articles in the final review was made based on a set of predetermined assessment criteria. These criteria entailed that articles would be excluded from the final review if: (1) the full-text of the article was not available in English; (2) the article was a duplicate or a different version of another article in the pool of articles; (3) interrelationships and links between Covid-19 pandemic, livestock systems and food security were not addressed; and (4) the main focus of the article is not on a developing country or a region. Based on these criteria, this full-text screening process reduced the results down to 68 articles that were included in this SLR, representing approximately 3% of the first-phase results (after duplicate removal) and 84.5% of the articles yielded from the title and abstract screening phase. Table S2 in the Supplementary Materials provides a list of articles analyzed of this SLR.

### 2.4. Article Coding and Data Extraction

In line with the aim of the SLR and the research questions, a coding scheme was developed in Microsoft Excel to extract relevant information from the literature elements finally included in the review. The structure and fields of the extraction form were iteratively adjusted and refined through a pilot extraction process. Extraction of this information was done by an author of this study and discussed with other co-authors. Each article in the coding scheme was given a unique ID, and the following metadata were extracted: title; authors’ names and affiliation details; year and month of publication; journal name; journal area; and research collaboration. In addition, we summarized the main research questions and key findings and categorized the articles based on the following aspects: livestock production system and animal type; scale of production; challenges other than COVID-19 to livestock systems; location/setting of research; the research type; the research design and methodology; sampling techniques; and the sample size, if any. Next, we performed a deeper content-based coding focusing on the two main dimensions of this study: livestock systems and food security. With regard to livestock systems, we explored the investigated stages and actors of the livestock chains, the phased of resilience addressed and the temporal dimension of COVID-19 impact (immediate, short-run, medium-run or long-run) investigated. In relation to food security, we coded the dimension of food security as well as the perspective taken in the article.

## 3. Results and Synthesis of Evidence

### 3.1. Descriptive Analysis of the Reviewed Articles

#### 3.1.1. Publication Characteristics

**Figure 2:**
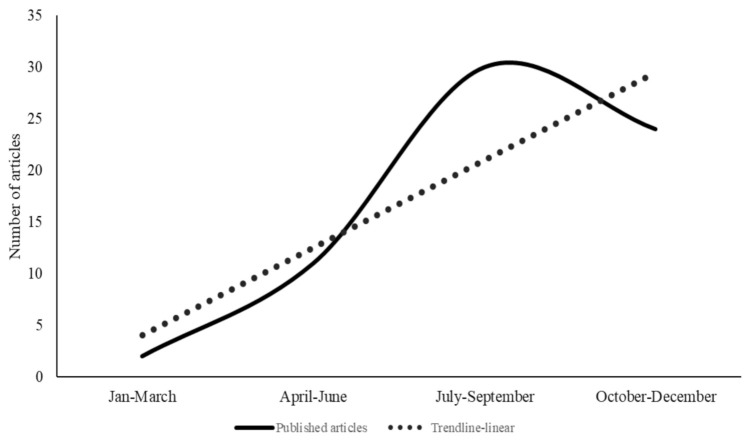
Number of the published articles included in the SLR sorted by the month of publication in 2020.

Figure 2 visualizes trends in the publication frequency of the surveyed literature during 2020, where all, except two, the articles in the review were published. A clear majority of around 63% of the surveyed literature (*n* = 43) was published in the form of ‘original articles’, whereas 28% (*n* = 19) of them were opinion articles, 6% were ‘review articles’ (*n* = 4) and 3% were letters to the editor (*n* = 2). During the first quarter of 2020 (January–March), only two articles linking COVID-19 pandemic to livestock systems and food security in developing countries were published. Through the second quarter and into the third quarter of 2020, the number of publications rose, peaking sharply in July and then in October with 18 papers each. The number of articles published during these two quarters constitutes about 60% of the papers in this study. From October through to December, 24 articles were published. 

While the first article in this review was published in December 2019, this indicates an increasing interest in this subject as well as the immediacy in attention and response of the scientific community to the outbreak of COVID-19 and its consequences on livestock systems and food security in developing countries. Furthermore, the frequency of these publications indicates an increased interest by researchers and practitioners in the effects of nature-induced risks on livestock systems in food security in these countries, while research on threats to agricultural systems in developing countries has traditionally centered on ‘crop’ production systems and viewed ‘livestock’ systems as an adjunct to crops [30]. Another important feature of this literature is the high level of international collaboration, where 70% of the reviewed articles involved authors affiliated with institutions in more than one country.

#### 3.1.2. Geographical and Spatial Distribution of Studies

**Figure 3:**
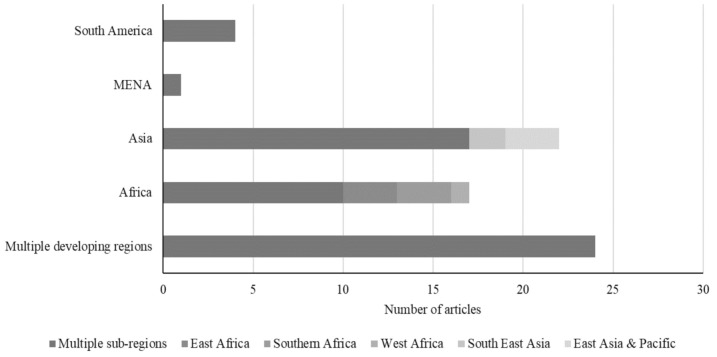
Distribution of the surveyed literature by the geographic region investigated.

As shown in Figure 3, around 35% of the reviewed articles (*n* = 24) reported findings from multiple regions of the developing world (e.g., developing countries, global south). In contrast, the remaining articles (65%) reported findings from specific developing regions. Of these, 22 articles reported findings from Asia, which consisted of 3 articles covering East Asian and Pacific countries, 2 articles covering South-East Asian countries and 17 articles covering multiple sub-regions within Asia. Another 17 studies reported findings from Africa, including 10 articles focusing on multiple sub-regions in Africa, 3 articles covering East African countries, 3 articles covering Southern African countries and one article focusing on West Africa. Together, articles reporting findings from Asian and African countries represent slightly more than two-thirds of the reviewed articles. This may be explained by the facts that these two continents are home to the most food insecure in the world and that livestock production contributes significantly to the livelihood and food security of many segments of their populations [26]. The rest of the reviewed articles reported findings from South America (4 articles) and only one article reported findings from North Africa and the Middle East (MENA) region. As shown in Table S3 in the Supplementary Materials, the most studied countries within reviewed literature are China and India, with each of them being the case study in eight reviewed articles, followed by Bangladesh and Kenya (two studies each).

From a spatial perspective, the results reveal that the surveyed literature addressed that COVID-19 affected livestock systems and food security both in urban and/or rural areas of developing countries. Specifically, 57% of the reviewed articles (*n* = 39) addressed the impacts of COVID-19 on livestock systems and food security in rural areas within developing countries, where agricultural activities and livestock production traditionally take place. Interestingly, the results show that around 51% of the articles (*n* = 35) focused on urban areas, which indicates the increased recognition of the role of urban livestock production in food security in developing countries. Finally, only 13% of the reviewed literature addressed the impacts of the pandemic on livestock systems and food security in peri-urban areas.

#### 3.1.3. Disciplinary Distribution and publication Outlets

According to the WOS classification of research areas (More details are here https://images.webofknowledge.com/images/help/WOS/hp_research_areas_easca.html, accessed on 26 February 2021), which consists of five broad categories, articles in this study can be grouped under two main research areas: ‘Life Sciences and Biomedicine’ (*n* = 49) and ‘Social Sciences’ (*n* = 12). Seven other articles were classified under other research areas. Within life sciences and biomedicine, the main research areas of the majority of studies were agricultural and veterinary sciences and infectious diseases (*n* = 16), environmental science and ecology (*n* = 15), food science and technology and nutrition (*n* = 5). In relation to social sciences’ category, the main research areas of the articles classified under this category were development studies, including gender studies (*n* = 8), biomedical social sciences (*n* = 8) and business and economics (*n* = 5). The most frequently used academic outlets for publication were the *Journal of Food Security* (10 articles), followed by *World Development*, *China Agricultural Economic Review*, *Frontiers in Veterinary Science*, and *International Journal of Environmental Research and Public Health*, with each of them being the publication outlet for 3 articles. With papers published in 50 different journals and combining diverse research sub-areas, this descriptive analysis suggests that the surveyed literature is disparate and multi-disciplinary and that disciplinary cooperation is constantly intensifying.

#### 3.1.4. Methodological Approaches and Investigated Livestock Systems

**Figure 4:**
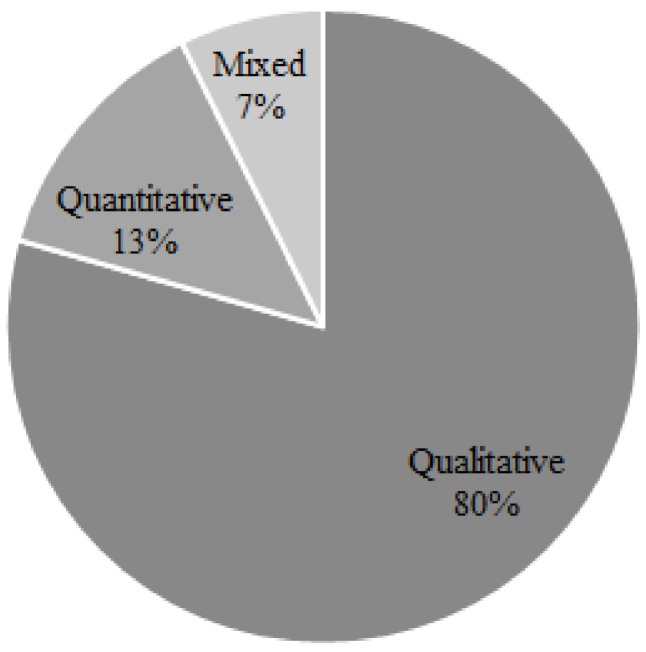
Distribution of the reviewed articles by the methodological approach.

Figure 4 shows that the methodological approach adopted in the reviewed literature was dominated by qualitative methods, whereas quantitative and mixed were used less frequently. A clear majority of the analyzed articles relied exclusively on literature (71%), whereas the remaining articles used several data collection tools, including secondary data (*n* = 6), interviews (*n* = 4) and questionnaires (*n* = 10). With respect to the investigated livestock systems, the results show that one-fourth of the reviewed articles analyzed specified multiple livestock systems (*n* = 18) and 10 articles did not specify the analyzed multiple production systems. Other articles (*n* = 40) focused on a specific production system, where poultry (*n* = 11), followed by pig (*n* = 9), fish (*n* = 6) and cattle (*n* = 4), were the most studied livestock production systems. Other production systems analyzed included goat, camel, sheep and buffalo. The majority of the reviewed literature (*n* = 33) focused on small-scale production systems, either farm- or household-based (*n* = 47), whereas 21 articles focused on commercial large-scale production systems. Generally, the focus of the reviewed literature on poultry systems and smaller scales of production may be attributed to the fact that small-scale poultry production is practiced by most rural households throughout the developing countries, particularly vulnerable groups including women. Thus, the potential of poultry systems, especially chicken systems, for supporting livelihoods and fostering food security of the poor segments of the population and achieving the goals of Agenda 2030 in many developing countries has been increasingly recognized by scholars and the development community [31]. 

An overall look at the methodological approaches and the livestock systems analyzed in the reviewed articles together with the scale of their production highlights clear symptoms of high aggregation and fragmentation of this literature, making it hard to draw generalizations about the interlinkages between Covid-19, livestock production, and food security. Therefore, it is unlikely that the existing literature can support the design of effective strategies and policy measures, as most of the articles do not consider the local contexts and socioeconomic landscapes.

### 3.2. Thematic and Content-Based Analysis of the Reviewed Articles

#### 3.2.1. Effects of the Pandemic on Livestock Supply Chains 

An obvious observation in the reviewed literature is that COVID-19 effects on livestock systems are often investigated in connection with other environmental stressors and sociodemographic and economic challenges (e.g., inequality and poverty) that traditionally influence the relationship between livestock systems and food security in developing countries. That is, the surveyed articles widely recognize that the extent to which Covid-19 impacts livestock systems and food security in a specific context depends on other environmental and socioeconomic stressors. For instance, Simula et al. [32] show that the pandemic magnified the already-existing effects of climate change on pastoralists and caused significant income losses and food insecurity challenges. Likewise, the literature recognizes that existing socioeconomic stressors (e.g., social inequalities, inter-state and regional conflict, population dynamics and economic instability) represent important explanatory factors that determine the impact of the pandemic on livestock systems and food security in developing countries. For instance, Mottaleb et al. [33] point out that COVID-19 has further exacerbated poverty and hunger in developing countries in several ways, including that the overburden on the healthcare sector may cause reallocation of resources to the healthcare sector and decrease resources allocated to agriculture and food sectors, thereby hampering livestock production and food security. 

**Figure 5:**
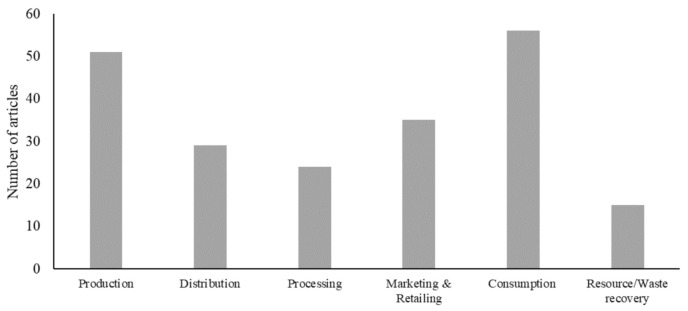
Distribution of the reviewed literature by the stage of livestock supply chain discussed.

Another observation in the reviewed articles is depicted by Figure 5, which shows that the pandemic has affected the functionality of both upstream and downstream stages of livestock value chains. That is, despite the fact that the disruptions caused by COVID-19 mainly affected transport, logistics and demand and consumption [34], effects on these downstream stages had a knock-back impact on producers and other upstream actors within livestock chains through short-run shocks to supply and demand in livestock markets [35]. However, according to Figure 5, the surveyed articles have unevenly addressed COVID-19 effects on the stages of livestock supply chains. In particular, the focus of the reviewed literature has mainly centered on livestock production (*n* = 51) and consumption (*n* = 56). However, other stages of the supply chain have received comparatively less attention: marketing and retailing (*n* = 31), distribution (*n* = 29) and processing (*n* = 27). In addition, post-consumption stages of the livestock supply chain received significantly less attention, with only 15 articles addressing aspects related to generation and management of livestock waste. The following paragraphs discuss the main stages of the livestock supply chain as addressed in the reviewed articles. 

**Livestock Production**: Pu and Zhong [36] show that restrictions on the movement between regions and social distancing measures have undermined production capacity of livestock commodities, decreased livestock production cycles and hindered producers’ access to production inputs. In particular, Kansiime et al. [37] reveal that livestock producers encountered significant challenges to access feed, fuel and vaccination procedures for reindeer and slaughtering facilities. As noted by Hossain et al. [38], the pandemic critically affected the dairy farming industry by increasing feed shortage, reducing accessibility to essential veterinary drugs and reducing consumer demand for dairy products. Together, these effects decreased the economic and productive efficiency of livestock producers and led to serious economic consequences on livestock producers in developing countries. In connection with these effects, Quayson et al. [39] highlight that small livestock producers in developing economies faced significant obstacles to access market information and extension services during the pandemic, which adversely affected their productivity and farm performance. Ejeromedoghene et al. [40] indicate that the inability of livestock producers to conduct rearing activities freely has affected both the welfare of farm animals and the livelihood of chain actors. Swinnen and McDermott [41] indicate that border restrictions on travel were especially costly for livestock producers who practice transhumance. Fan et al. [42] reveal that restrictions on live poultry trading led to live burials of chicken seedlings and that many producers faced challenges in relation to lack of inputs, especially feed, lack of labor due to quarantine, price volatility of feed and other inputs, which put breeding companies and livestock producers on the edge to bankruptcy. In the same context, Mottaleb et al. [33] highlight that informal workers and casual labor have been mostly affected by the pandemic-induced contraction of employment and movement restriction measures, which has led in many cases to labor shortages and massively disrupted livestock supply [43]. 

**Post-production, distribution and marketing activities**: The reviewed evidence shows that the pandemic reduced access of livestock producers to domestic markets [40,44]. In particular, restrictions on public transport constrained farmers’ access to input markets and output markets, as many of them transport inputs to the farm and production to markets by public transportation means [23]. Swinnen and McDermott [41] illustrate that wholesaling and logistics’ operations in trucking, which represent important means for transporting agrifood commodities in developing countries, were disrupted by restrictions on human mobility and on wholesale markets. Mishra et al. [35] and Ejeromedoghene et al. [40] point out that restricted international trade due to the large-scale restrictions in aviation activities and border closures lulled international marketing and export and import of livestock commodities and delayed the entry of imported livestock commodities to importing markets. 

**Retailing and food services’ industry**: The reviewed literature shows that the closures of restaurants and food services and logistic restrictions on tourism have severely affected actors involved in the marketing and retailing stages of the livestock supply chain in developing countries [22]. In particular, retailing has been reorienting towards online platforms, and more packaged, longer-life and processed meat and dairy products. For instance, USDA [45] shows that actors in livestock chains in Burma faced major challenges during the COVID-19 pandemic as trade slowed, consumption decreased and tourism stopped, causing increased surplus and decreased prices for livestock commodities. In Ethiopia, farmers were less able to sell milk and thus more milk was processed and the butter supply rose, leading to sharp decreases in butter prices in rural areas. FAO [11] indicates that the pandemic has reduced processing capacity of meat and dairy due to staff reduction constraints, challenges related to food transport and shifts in retailing and food consumption habits. Other elements in this literature argue that changes in consumer behavior due to social distancing and restaurant closures have increased online purchasing, which may seriously harm small- and medium-sized distributors and retailers, who do not often have web-based product and service delivery [46]. 

**Consumption of livestock-source foods**: Hobbs [47] and Galanakis [48] illustrate that COVID-19 led to panic buying and stockpiling behaviors, induced short-term changes in food consumption patterns by decreasing consumption away from home and increased consumption of basic foods such as eggs and food prepared at home. Therefore, Mishra et al. [35] predict that the demand for agricultural commodities, including livestock-source foods, may decline as institutions like restaurants tend to have a higher willingness-to-pay for produce. In addition, loss of incomes and increased rates of unemployment and under-employment have severely reduced the purchasing power of consumers, particularly those who were already on the borderline of poverty, and decreased their economic access and consumption of livestock-source foods [22,44]. Such changes in consumption patterns of livestock-source foods increase the pressure on the livestock supply chain and also have negative impacts on small-scale producers in developing countries [49]. For example, Biswal et al. [50] show that households switching from the consumption fresh milk to packaged milk caused a significant huge setback for small-scale farmers in both urban and rural areas in India.

**Livestock-source food’s loss and waste:** The reviewed articles addressed the effects of the pandemic on postharvest losses and wastage of livestock products. In this respect, Gregorio et al. [51] point out that reduced access of livestock producers to farm inputs and to consumer markets negatively affected on-farm productivity and increased wastage and post-harvest losses, particularly because of their relatively short shelf life. Farrell et al. [52] illustrate that closure of livestock markets and stalls decreased farmers’ ability to sell their produce, and thus increased food waste and affected farmers’ incomes and livelihood. Furthermore, Ma et al. [53] reveal that disruption of the logistical channels, shortage of farm labor, and drop in demand have decreased sales and lowered prices of livestock commodities, generating high post-harvest losses and forcing disposal of produce [50]. Overall, elements in this subset of the reviewed literature suggest that the pandemic has produced new challenges to livestock waste management at the production and consumption stages due to changes in consumption and waste disposal patterns and behaviors during the lockdown periods [1,54]. 

#### 3.2.2. Effects of the Pandemic on the Resilience of the Livestock Systems 

To further examine COVID-19 effects on livestock systems in developing countries, an additional coding process was undertaken using the National Research Council’s four-stage definition of resilience [55] (plan, absorb, recover and adapt) to identify and analyze the resilience criteria addressed in the reviewed articles. According to this definition, resilience is ‘the ability to *prepare* and *plan* for, *absorb*, *recover* from, and more successfully *adapt* to adverse events’. From a supply chain management perspective, building resilience of a complex supply chains, such as livestock supply chains, to cope with COVID-19 effects and better prepare for projected future pandemics and extreme events entails enhancing the ability of supply chain actors to anticipate, adapt to, respond to, recover from and learn from disruptions [56,57]. 

**Figure 6:**
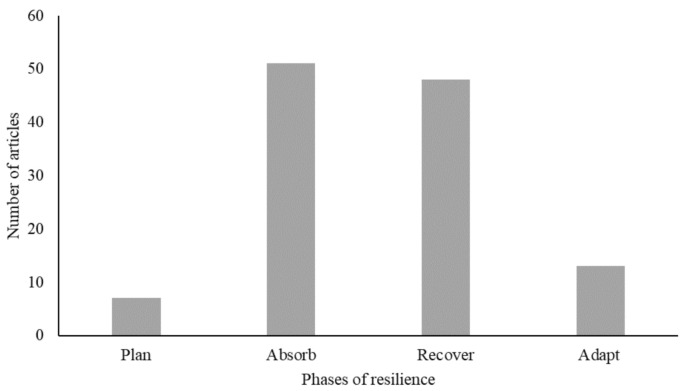
A breakdown of the reviewed literature by the phase of resilience addressed.

Figure 6 shows the phases of livestock systems’ resilience that were addressed in the reviewed literature. Notably, many articles investigated the resilience of livestock supply chains to the COVID-19 pandemic without explicitly defining resilience, and only few of them discussed all four phases of resilience. Specifically, Figure 6 indicates that the majority of the reviewed articles focused on the ‘absorbance’, i.e., the capacity of livestock systems to limit the impact of the COVID-19 pandemic (*n* = 51), and the ‘recovery’, i.e., the ability of livestock systems to return to their original state following the pandemic (*n* = 48), of livestock systems. Articles cited in the previous sub-section of this paper belong mainly to these two phases of resilience, where the focus has been on how livestock systems were affected by- and responded to the COVID-19-induced risks, such as decreased farm productivity, reduced access to consumer and input markets, shortage of farm labor, reduced consumers’ purchasing power and vulnerability of market prices, to cite a few [1,22,35,40,58]. Based on the investigation of these risks and their effects, the reviewed literature turns then to focus on immediate and short-run countermeasures implemented mainly by governments and actors within livestock supply chains to mitigate the impact of the pandemic and restore the ability of livestock systems to return to their original state and resume livestock activities along the supply chain [40,59,60,61].

In contrast, a smaller subset of the reviewed literature focused on action taken by the supply chain actors to ‘plan’ (*n* = 7) or to ‘adapt’ (*n* = 13) livestock systems to reduce their vulnerability, improve their learning from the pandemic and enhance their resilience to observed and anticipated pandemics and disasters. In this regard, Linkov and Trump [62] indicate that increasing uncertainty and complexity in global (livestock) systems entail addressing the role of preparedness and recovery from disruption, as neglecting these aspects may lead to a limitation in the understanding of how livestock supply chain can maintain resilience against the COVID-19 and future pandemics. Furthermore, elements of this subset of the surveyed literature emphasize the importance of addressing the broader sustainability challenges, and the need to ensure the functionality of domestic and regional livestock markets and transform sustainably livestock sectors in developing countries to build resilient systems with higher preparedness and capacity to anticipate and adapt to new challenges and burgeoning natural and environmental risks [42,63,64,65,66].

#### 3.2.3. Interlinkages between Covid-19 Impact of Livestock Systems and Food Security

**Figure 7:**
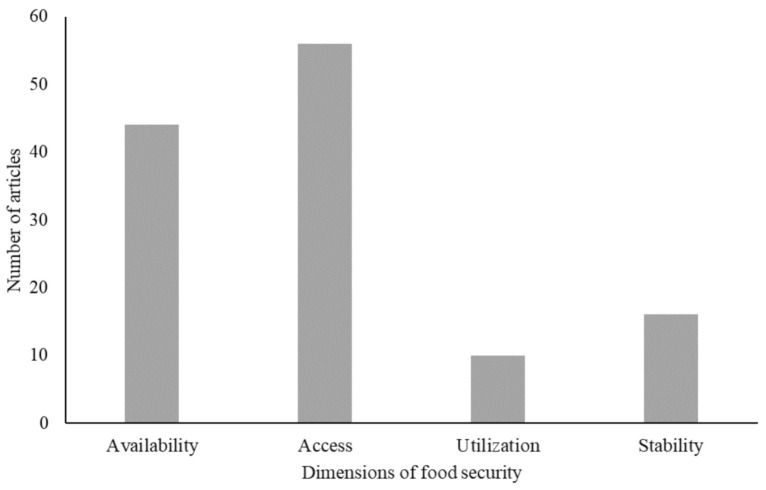
Breakdown of the reviewed literature by the dimensions of food security addressed.

Figure 7 indicates that the reviewed literature unevenly recognizes the links between COVID-19 effects livestock systems and different dimensions of food security: ‘accessibility’ (*n* = 58), ‘availability’ (*n* = 46), ‘utilization’ (*n* = 10) and ‘stability’ (*n* = 16). The special focus of the articles included in this review on accessibility to livestock-source food and food security in the context of COVID-19 pandemic is attributable to the unique nature of this pandemic, which differs from previous pandemics in terms of both origins and pathways of impact on livestock systems and food security [21,22]. That is, unlike previous disease outbreaks in recent decades, COVID-19 did not directly affect the production stage (availability dimension) of livestock supply chains [21]; however, it disrupted transport and logistics, labor markets as well as consumer demand for livestock products, making accessibility to food the major dimension of impact. Thus, several studies point out that the pandemic has increased challenges related to food accessibility among poor households both in rural and urban areas in developing countries due to interconnected factors related to different nodes of livestock supply chain together with rising livestock prices and decreasing households’ incomes and purchasing power. In addition, the literature suggests that the pandemic has already and is likely to reduce households’ access to livestock commodities and push more non-poor households to fall into the category of vulnerable and food insecure population [67]. In particular, the literature points out that the effects of the pandemic on food security will be severer for women, children and other vulnerable population groups [37,52]. Therefore, several authors argue that the impact of COVID-19 on food security in developing countries would have broader magnitude as it affects a wider range of stages of livestock chains, multiple actors along these chains and several segments of the population [68,69]. In the next paragraphs, we briefly summarize the main issues that the surveyed literature addressed in relation to each of the four dimensions of food security.

With regard to ***availability*** of livestock commodities, the literature attributes food insecurity to inadequacy of on-farm disease surveillance, shortage of feed ingredients and veterinary medications, closure of processing facilities, restrictions on trade of livestock inputs and commodities, increased costs for feeds and medication, and shortage of farm labor [22,35,38,40]. Collectively, these effects adversely influenced livestock production and productively and decreased availability of livestock-source foods in various markets. In relation to ***accessibility*** to livestock-source foods, the literature attributes food insecurity effects to both physical and economic accessibility causes since a large proportion of people in developing countries have fragile livelihoods and depend on informal livestock and other agricultural supply chains, making them more vulnerable to COVID-19 effects. The physical causes include problems with logistics restrictions in relation to transportation, distribution and delivery, and closure of primary and secondary livestock markets [35,53,70]. The economic accessibility to food was linked to layoffs of farm labor, unemployment, loss of incomes and livelihoods, reduced purchasing power, and reduced remittances [51,52,63,71]. The magnitude of these issues related to accessibility to livestock-source food was linked to the duration of the pandemic’s containment measures, that is, the literature indicates that the longer these lockdown policies and other containment measures are in place, the more challenging the recovery process will be since they will particularly affect vulnerable populations, exacerbate pre-existing inequities and generate further food security challenges [38,72].

Concerning the ***utilization*** dimension, the literature briefly touched on COVID-19 effects on people’s ability to utilize the food (e.g., store, cook, prepare and share) in ways that could have positive nutritional impacts. For example, Farrell et al. [52] highlight the likely hygiene and food safety effects of the pandemic on consumers in developing countries due to the informal nature of food services, which represent a significant share of the food industry in these countries, and the limited storage capacities for fresh livestock foods. Furthermore, Laborde et al. [73] and Kansiime et al. [70] illustrate that the pandemic may influence consumers’ dietary choices in the short-run and cause shifts in consumer demand toward cheaper and less nutritious foods, which threatens to worsen food security outcomes in developing countries. Lastly, a common observation across the reviewed literature addressing the ***stability*** of supply and demand for livestock commodities is that measures suggested in response to COVID-19 effects on food security revolved around immediate, direct and short-run interventions, such as the provision of safety nets and food assistance programs [71,74], whereas few articles looked at the long-term impacts of the pandemic on livestock supply chains and their consequences on food security and nutrition outcomes. This could be attributed to the fact that we know very little about the future dynamics of this pandemic, especially that even many of its short-run effects were unprecedented and hard to predict. What is clear, however, is that if the livestock system does not change, more people will suffer from food insecurity and we will be unprepared when the next global emergency hits. Therefore, addressing the effects of COVID-19 on different dimensions of food security both in the short- and long-run can help put developing countries and the global livestock system on a resilient path and mitigate the food insecurity outcomes of future pandemics and shock events.

## 4. Discussion and Implications for Future Research

The review confirms that the outbreak and consequences of the COVID-19 pandemic have posed significant stresses to livestock systems in developing countries and drastically disrupted many activities along livestock supply chains. Despite this, many of these disruptions are temporary and have short-run effects since they have resulted from measures adopted by governments in developing countries to contain the spread of the virus. Our results imply that livestock systems represent a main ‘intermediary’ pathway through which shock events, such as the COVID-19 pandemic, can be transmitted to affect various dimensions of food security. This is because many segments of the population in developing countries rely on livestock chains for their livelihood and food consumption. From a scientific research perspective, the results of this systematic literature review point towards a number of research gaps, which underscore the need for further studies to enhance the understanding of how livestock systems might respond to, recover from and adapt to this and future pandemics in order to meet food security objectives.

First, although research response to COVID-19 impact on livestock systems, and on food security in developing countries, has been rapid in terms of both the quantity and temporal succession, the results present a highly suggestive disjunction in studies analyzing the interactions between COVID-19 pandemic, livestock systems and food security in developing countries. This is exemplified by the existence of rich and growing bibliography on COVID-19 and each of the other two components taken in isolation (that is, COVID-19 and livestock system or COVID-19 and food security). However, the number of published articles diminishes significantly when considering the interlinkages between the three of them. That is, only 68 articles of the 2681 candidate articles identified after initial screening and duplicate removal were included in the qualitative synthesis. Another example showing the fragmentation in the reviewed articles is that only very few of the reviewed articles referred to the ‘one health approach’ in the context of understanding COVID-19 impact on livestock systems and food security in developing countries, and none them used ‘one health’ as a keyword (see Figure S1 in the Supplementary Material). This was quite surprising given that the one health approach has been widely recognized in recent years as a versatile cross-disciplinary approach to incorporate human, animal and environmental health in order to solve complex problems, such as infectious disease outbreaks [75,76]. This emphasizes the need for more holistic approaches to recognize the complex nature of livestock chains in developing countries and address the multifaceted and wide-spread effects of COVID-19 on food security. Such holistic research approaches would offer opportunities to take a system perspective on all livestock-related activities, including input supply, production, processing, transportation, marketing, distribution and consumption. This would allow for comprehensive analyses of the impact of COVID-19 pandemic in relation to other drivers (e.g., biophysical, technological, demographical and socio-economic) that influence livestock supply chains and food demand. Then, this can provide a framework to link these drivers to more immediate factors affecting food and nutrition security at the household level (e.g., availability, accessibility and safety of livestock-source food), and thus understand possibilities for improving food access and ensuring equity across livestock systems for producers, intermediaries and consumers of livestock-source food.

Second, the existing literature in relation to the interrelationship between COVID-19, livestock systems and food security in developing countries is dominated by exploratory and qualitative studies, which tend to describe the effects of the pandemic on livestock systems and food security in different contexts of developing countries rather than measuring the extent and magnitude of these effects. This is a typical feature of a new and emerging literature, but future research undertakings should focus more on how to use the findings of these exploratory and qualitative studies to strengthen the theoretical foundation of research on pandemics and infectious diseases, livestock systems and food security, which can then inform theme creation for specific quantitative investigations. In the same context, the results show also that the reviewed literature is regionally and continently focused and lacks a connection to specific countries within developing countries. Regional and continental estimates of COVID-19 impact on livestock supply chains and food security do not take into consideration the unique situations of individual developing countries in terms the specific characteristics of livestock value chains and the magnitude of COVID-19 on food security and nutrition, making it impossible to draw generalizations under conditions of such heterogeneity. In this respect, there is a large body of literature demonstrating that data gaps are an inherent part of livestock systems in developing countries and highlighting the need for disaggregated country-level data and research by livestock system to assist in identifying and measuring the impact of various stressors on livestock systems and actors involved [77,78].

Third, the results reveal that the literature tends to focus on the production and consumption stages of livestock supply chains in developing countries, whereas other stages and actors along these chains (e.g., distributers, processors and retailers) receive comparatively less attention. Traditionally, research on livestock supply chains in developing countries has always perceived intermediaries as opportunist parasites who take advantages of livestock producers’ unawareness of market price and their weak bargaining power [79]. However, intermediaries play vital roles in livestock value chains in transferring livestock products from farm gates to consumers, increasing added value of agrifood commodities by performing grading, packaging and processing activities, and providing marketing services to small producers who would bear high transaction costs if they had to perform these activities [80]. According to Deepak et al. [71], such negative perception seems to be reflected in agricultural policies and academic publications in relation to developing countries, which widely ignore the traders and intermediaries, creating a ‘missing middle’ in research endeavors in relation to livestock systems and food security in developing countries [5]. The lack of research that examines the propagation of shocks through livestock supply chains limits our understanding of the true effects of COVID-19 on the livestock systems as a whole and the subsequent effects on food security. As noted by Davis et al. [81], the interconnectedness of food systems entails that a shock that influences any stage of the supply chain will undoubtedly affect subsequent stages. Therefore, more consistent research endeavors are needed to develop better understanding of the dynamics of propagation of COVID-19 impacts across livestock supply chains in order to inform adequate interventions that can enhance the performance and sustainability of these chains and mitigate the food insecurity outcomes.

Fourth, another major finding in this review is related to the resilience of livestock value chains to risks posed by the COVID-19 pandemic. The majority of the reviewed articles focused on the ‘absorbance’ and ‘recovery’ phases of resilience, whereas only a few articles addressed actions taken by the supply chain actors to ‘plan’ or to ‘adapt’ livestock systems to reduce their vulnerability and enhance their learning from and resilient to the COVID-19 pandemic. This suggests a lack of awareness of the important role of these criteria in resilience building and risk management settings. While projections indicate that pandemics will be more frequent events in the future [20], future research should not only seek to reduce the effects and vulnerability of livestock systems in developing countries but also to foster their preparedness and adaptive capacity to future pandemics and potential risks, particularly the barriers and enablers that determine their ability to adapt and recover from such events. Such integrated resilience-based approaches are crucial in order to take on effective preventive measures before supply chain disruption and recovery measures after disruptions have occurred.

Fifth, we find that the consequences of the COVID-19 pandemic on food security are typically defined narrowly with a special focus on the ‘availability’ of livestock commodities and ‘accessibility’ to livestock-source foods. Considerably less attention was given to utilization and stability dimensions of food security. Furthermore, lesser attention was given to the discussion of the two additional dimensions (agency and sustainability) that have recently been suggested and have become increasingly recognized as dimensions to achieving food security and sustainable food systems [82]. In this respect, Davis et al. [81] point out that a shock, such as the COVID-19 pandemic, can generate differential effects on various dimensions of food security, which emphasizes the need to expand our understanding of the impact of shock events beyond their effects on production and food availability. An acknowledgement of all dimensions that drive food security and their interconnections is crucial in order to minimize systemic risks and enhance the capacity of developing countries to build resilience of livestock systems against future pandemics that can help achieve food security objectives [83].

Last, despite our attempt to provide a comprehensive review of the literature, this SLR has some limitations. First, we recognize that this body of the literature is quite recent (since December 2019) and that many research outputs on this topic might be in the pipeline, including articles that are already under review by the journals and conference publications. Second, despite the contribution that popular science and grey literature could make to this SLR in terms of adding to the understanding of how COVID-19 pandemic affected livestock systems and food security, we excluded these sources and exclusively reviewed research published in peer-reviewed journals and academic outlets contained in the three identified databases. Third, our review only included peer-reviewed articles published in English, which makes it likely that we missed important articles published in other languages. Nevertheless, we argue that the bibliographic databases’ search strategy was comprehensive and was designed in a way that generated articles based on multiple keywords and their synonyms. In addition, the inclusion/exclusion criteria were defined rigorously, and the processes of coding, extracting and information from the identified articles and their synthesis were implemented with proper validation and quality assurance.

## Data Availability

The data presented in this study are available on request from the corresponding author.

## Supplementary Materials

The following are available online at https://www.mdpi.com/article/10.3390/pathogens10050586/s1, Table S1. Search codes used in the bibliographic databases’ search, Table S2. List of the reviewed literature, Table S3. Studied countries and cities in the reviewed literature, and Figure S1. Keywords used in the reviewed articles as identified by authors.
