# Supplementary material for: COVID-19, Livestock Systems and Food Security in Developing Countries: A Systematic Review of an Emerging Literature
Source: Pathogens. 2021 May 11;10(5):586. doi: 10.3390/pathogens10050586 (PMC8151861; doi:10.3390/pathogens10050586)

Supplementary Material

COVID-19, Livestock Systems & Food Security in Developing Countries: A systematic review of an emerging literature

Table S.1. Search codes used in the bibliographic databases' search

1. Covid-19 (n= 11)
Phenomenon: covid OR covid-19 OR coronavirus OR corona virus OR coronavirus disease OR covid-19 pandemic OR ncov OR novel coronavirus OR sarscov OR sars-cov OR 2019ncov.
2. Livestock Systems (n= 44)
abattoir OR (animal AND system OR disease, health, welfare, products) OR butchers OR commercial OR cooperatives OR distribution OR farmer OR feed OR fodder OR herders OR inputs OR (livestock AND commodities OR keeper, system, products) OR labor OR labour OR marginalized OR market OR market access OR organization OR pastoralist OR pasture OR processing OR producer OR production OR retailer OR rural OR slaughter OR small-scale OR small-and medium sized OR smallholder OR subsistence OR supply chain OR sustenance OR transportation OR vaccination OR value chain OR wage OR waste.
3. Food Security (n= 36)
animal sourced food OR diet OR dietary diversity OR empowerment OR energy-dense foods OR food OR food security OR food access OR food availability OR food consumption OR food demand OR food equality OR food inequality OR food insecurity OR food prices OR food production OR food quality OR food safety OR food security OR food sovereignty OR food stability OR food supply OR food utilization OR hunger OR income OR livelihood OR livestock-source food OR macronutrients OR malnutrition OR micronutrients OR micronutrients deficiency OR nutrition OR poverty OR stunting OR undernourishment OR undernutrition.
4. Developing countries (n= 92)
Developing countries OR global south OR low-income OR low and middle income countries OR Africa OR Algeria OR Angola OR Asia OR Bangladesh OR Barbados OR Benin OR Botswana OR Brunei OR Burkina Faso OR Burundi OR Cabo Verde OR Cameroon OR Caribbean OR Central Africa OR Central America OR Chad OR China OR Comoros OR Congo OR Costa Rica OR Côte d'Ivoire OR Cuba OR Democratic Republic of Congo OR Djibouti OR Dominican republic OR East Africa OR East Asia OR Egypt OR El Salvador OR Equatorial Guinea OR Eritrea OR Ethiopia OR Gabon OR Gambia OR Ghana OR Guatemala OR Guinea OR Guinea-Bissau OR Guyana OR Haiti OR Honduras OR Hong Kong OR India OR Indonesia OR Iran OR Jamaica OR Kenya OR Latin America OR Lesotho OR Liberia OR Libya OR Madagascar OR Malawi OR Mauritania OR Mauritius OR Mexico OR Morocco OR Mozambique OR Myanmar OR Namibia OR Nepal OR Nicaragua OR Niger OR Nigeria OR North Africa OR Pakistan OR Panama OR Papua New Guinea OR Philippines OR Republic of Korea OR Rwanda OR Sao Tome and Principe OR Senegal OR Sierra Leone OR Singapore OR Somalia OR South Africa OR South America OR South Asia OR Southern Africa OR Sri Lanka OR Sudan OR Taiwan OR Thailand OR Togo OR Trinidad and Tobago.

Table S.2. List of the reviewed literature

Title		Year	Authors
1.	A critical review of the impacts of COVID-19 on the global economy and ecosystems and opportunities for circular economy strategies	2021	Ibn-Mohammed T. et al.
2.	Aid to Africa's agriculture towards building physical capital: Empirical evidence and implications for post-COVID-19 food insecurity	2020	Asiedu E. et al.
3.	Averting hunger in sub-Saharan Africa requires data and synthesis	2020	Porciello J. et al.
4.	Compound natural and human disasters: Managing drought and COVID-19 to sustain global agriculture and food sectors	2021	Mishra A. et al.
5.	COVID-19 implications on household income and food security in Kenya and Uganda: Findings from a rapid assessment	2021	Kansiime M.K. et al.
6.	COVID-19 induced economic loss and ensuring food security for vulnerable groups: Policy implications from Bangladesh	2020	Mottaleb K.A. et al.
7.	COVID-19, wet markets, and planetary health	2020	Petrikova I. et al.
8.	Digital Inclusion for Resilient Post-COVID-19 Supply Chains: Smallholder Farmer Perspectives	2020	Quayson M. et al.
9.	Ecology and economics for pandemic prevention: Investments to prevent tropical deforestation and to limit wildlife trade will protect against future zoonosis outbreaks	2020	Dobso A.P. et al.
10.	Food insecurity will be the sting in the tail of COVID-19	2020	The Lancet Global Health
11.	Food security and safety concerns in animal production and public health issues in Africa: A perspective of COVID-19 pandemic era	2020	Ejeromedoghene O. et al.
12.	Growing and eating food during the COVID-19 pandemic: Farmers' perspectives on local food system resilience to shocks in Southern Africa and Indonesia	2020	Paganini N. et al.
13.	Hand in Glove? Processes of Formalization and the Circular Economy Post-COVID-19	2020	Dewick P. et al.
14.	How Africa Is Promoting Agricultural Innovations and Technologies amidst the COVID-19 Pandemic	2020	Joseph Fernando A.
15.	Informal food chains and agrobiodiversity need strengthening—not weakening—to address food security amidst the COVID-19 crisis in South America	2020	Zimmerer K.S. & de Haan S.
16.	Rising concerns over agricultural production as COVID-19 spreads: Lessons from China	2020	Pu M. & Zhong Y.
17.	Sustainable intensification of agriculture in the context of the covid-19 pandemic: Prospects for the future	2020	Sampath P.V. et al.
18.	The gendered impacts of COVID-19 amidst agrarian distress: Opportunities for comprehensive policy response in agrarian South Asia	2020	Nichols C.E. et al.
19.	The influence of COVID-19 on agricultural economy and emergency mitigation measures in China: A text mining analysis	2020	Pan D. et al.
20.	Viewpoint: The future of work in agri-food	2020	Christiaensen L. et al.
21.	Will novel coronavirus (COVID-19) pandemic impact agriculture, food security and animal sectors? [Qual será o impacto da pandemia do novo coronavirus (COVID-19) na agricultura,	2020	Seleiman M.F. et al.
22.	An assessment of socioeconomic impact of COVID-19pandemic in India	2020	Aneja, R &Ahuja, V.
23.	Assessing the Impact of the COVID-19 Pandemic on Agricultural Production in Southeast Asia: Toward Transformative Change in Agricultural Food Systems	2020	Gregorioa, G.B. & Ancog, R.C.
24.	Beyond banning wildlife trade: COVID-19, conservation and development	2020	Roe, D. et al.
25.	Beyond the Disease: Contextualized Implications of the COVID-19 Pandemic for Children and Young People Living in Eastern and Southern Africa	2020	Govender, K. et al.

Title	Year	Authors
26. Challenges for Indonesia Zero Hunger Agenda in the Context of COVID-19 Pandemic	2020	Paramashanti, B.A.
27. Could Covid-19 Worsen Food Insecurity in Burkina Faso?	2020	Zidouemba, P.R. et al.
28. COVID-19 and Pacific food system resilience: opportunities to build a robust response	2020	Farrell, P. et al.
29. Globalisation in the time of COVID-19: repositioning Africa to meet the immediate and remote challenges	2020	Yaya, S. et al.
30. How Indian agriculture should change after COVID-19	2020	Kumar, A. et al.
31. How to prevent a global food and nutrition security crisis under COVID-19?	2020	Fan, S.G. et al.
32. Impact of COVID-19 and associated lockdown on livestock and poultry sectors in India	2020	Biswal, J. et al.
33. Impact of COVID-19 on China's agricultural trade	2020	Cao, L.J. et al.
34. Impacts of COVID-19 on the Agri-food Sector: Food Security Policies of Asian Productivity Organization Members	2020	Hossain, S.T.
35. Impacts of the COVID-19 Pandemic on the Global Agricultural Markets	2020	Elleby, C. et al.
36. Informal food traders and food security: experiences from the Covid-19 response in South Africa	2020	Wegerif, M.C.A.
37. Key indicators for monitoring food system disruptions caused by the COVID-19 pandemic: Insights from Bangladesh towards effective response	2020	Amjath-Babu, T.S. et al.
38. On the susceptibility and vulnerability of agricultural value chains to COVID-19	2020	Morton, J
39. Perception of health risks in Lao market vendors	2020	Philavong, C. et al.
40. Resilient agri-food systems for nutrition amidst COVID-19: evidence and lessons from food-based approaches to overcome micronutrient deficiency and rebuild livelihoods after crises	2020	Heck, S. et al.
41. Strategic assessment of COVID-19 pandemic in Bangladesh: comparative lockdown scenario analysis, public perception, and management for sustainability	2020	Shammi, M. et al.
42. The care economy and the state in Africa's Covid-19 responses	2020	Ossome, L.
43. The impact of epidemics on agricultural production and forecast of COVID-19	2020	Zhang, S.R. et al.
44. What is the impact of COVID-19 disease on agriculture?	2020	Siche, R.
45. Zoonotic Pathogens of Dromedary Camels in Kenya: A Systematised Review	2020	Hughes, E.C. & Anderson, N.E.
46. COVID-19 and pastoralism: reflections from three continents	2020	Mohamed, T.S. et al.
47. COVID-19 in pastoral contexts in the greater Horn of Africa: Implications and recommendations	2020	Griffith, E.F. et al.
48. COVID-19 Lockdown, Food Systems and Urban-Rural Partnership: Case of Nagpur, India	2020	Sukhwani, V. et al.
49. COVID-19, Systemic Crisis, and Possible Implications for the Wild Meat Trade in Sub-Saharan Africa	2020	Mamun, M.A. & Ullah, I.
50. Economic and Social Impacts of COVID-19 on Animal Welfare and Dairy Husbandry in Central Punjab, Pakistan	2020	Hussain, S. et al.
51. Energy Engineering Approach for Rural Areas Cattle Farmers in Bangladesh to Reduce COVID-19 Impact on Food Safety	2020	Nur-E-Alam, M. et al.
52. Food system disruption: initial livelihood and dietary effects of COVID-19 on vegetable producers in India	2020	Harris, J. et al.
53. Generational Differences in Perceptions of Food Health/Risk and Attitudes toward Organic Food and Game Meat: The Case of the COVID-19 Crisis in China	2020	Xie, X.R. et al.

Title		Year	Authors
54.	10 recommendations for African governments to ensure food security for poor and vulnerable populations during COVID-19	2020	Lawson-Lartego L & Cohen M.J.
55.	A framework for identifying and mitigating the equity harms of COVID-19 policy interventions	2020	Glover R.E. et al.
56.	Food Sovereignty of the Indigenous Peoples in the Arctic Zone of Western Siberia: Response to COVID-19 Pandemic	2020	Bogdanova E. et al.
57.	How is Brazil facing the crisis of Food and Nutrition Security during the COVID-19 pandemic?	2020	Carvalho C.A. et al.
58.	Mapping disruption and resilience mechanisms in food systems	2020	Savary S. et al.
59.	Middle East respiratory syndrome coronavirus infection in non-camelid domestic mammals	2019	Kandeil A. et al.
60.	Perspectives from CO+RE: How COVID-19 changed our food systems and food security paradigms	2020	Bakalis S. et al.
61.	Planetary Food Commons and Postcapitalist Post-COVID Food Futures	2020	Healy S. et al.
62.	Research Progress on Coronavirus Prevention and Control in Animal-Source Foods	2020	Gan Y. et al.
63.	Strengthening Local Food Systems in Times of Concomitant Global Crises: Reflections From Chile	2020	Kanter R. & Boza S.
64.	Animal Welfare and Livestock Supply Chain Sustainability Under the COVID-19 Outbreak: An Overview	2020	Hashem N.M. et al.
65.	Animal-based food systems are unsafe: severe acute respiratory syndrome coronavirus 2 (SARS-CoV-2) fosters the debate on meat consumption	2020	Jacob M.C.M. et al..
66.	Challenges to the Poultry Industry: Current Perspectives and Strategic Future After the COVID-19 Outbreak	2020	Hafez H.M. & Attia Y.A.
67.	Conceptualising COVID-19's impacts on household food security	2020	Devereux S. et al.
68.	Covid-19 pandemic in the lens of food safety and security	2020	Ma N.L. et al.

Table S.3. Studied countries and cities in the reviewed literature

Countries	No. of Articles	Cities	No. of Articles
Kenya	4	Maputo	1
Uganda	2	Toraja	1
Bangladesh	4	Java	1
Ghana	1	Cape Town	1
Zimbabwe	2	Masvingo	1
Mozambique	1	Lima	1
Indonesia	2	Arequipa	1
Peru	1	Cusco	1
China	8	Huancayo	1
India	8	Huánuco	1
Nepal	1	Nagpur	1
Brazil	2	Punjab	1
Thailand	1	Sharkia	1
Taiwan	1	Beheira	1
Burkina Faso	2	Luxor	1
Ethiopia	2		
Morocco	1		
Laos	1		
Somalia	1		
Eritrea	1		
Djibouti	1		
Sudan	1		
Tanzania	1		
Pakistan	1		
Siberia	1		
Egypt	1		
Chile	1		
Saudi-Arabia	1		
Unspecified	14	Unspecified	53
Total	68	Total	68

Figure S.1. Keywords used in the reviewed articles as identified by authors

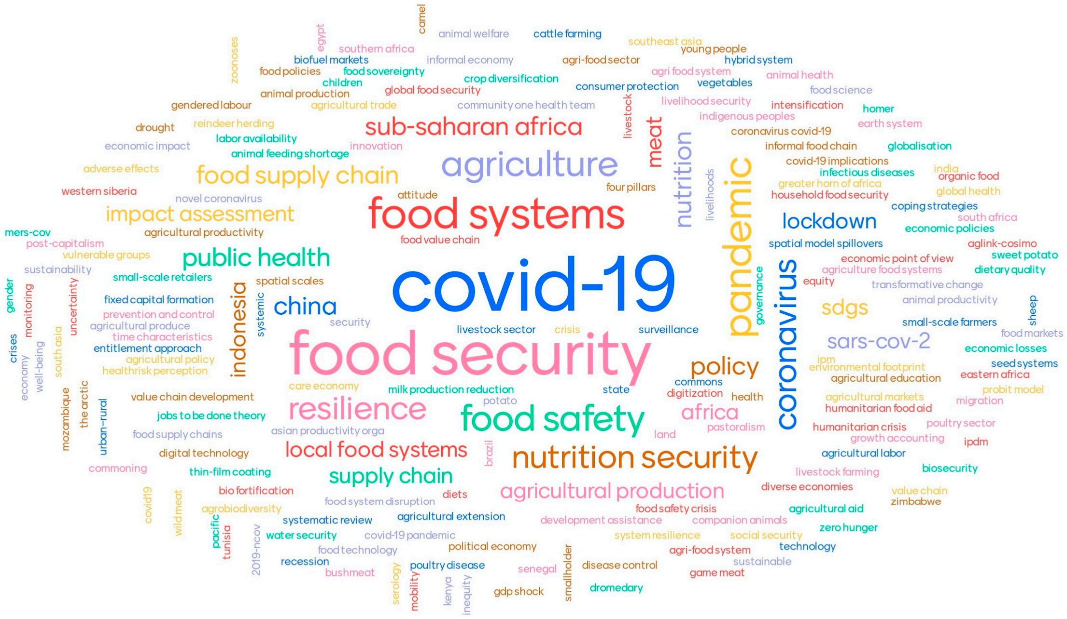

Supplement: Supplementary file 1 [file pathogens-10-00586-s001.zip › pathogens-1197876-supplementary.pdf]
